# A qualitative study in rural and urban areas on whether – and how – to consult during routine and out of hours

**DOI:** 10.1186/1471-2296-7-26

**Published:** 2006-04-26

**Authors:** Neil C Campbell, Lisa Iversen, Jane Farmer, Clare Guest, John MacDonald

**Affiliations:** 1 Department of General Practice and Primary Care, University of Aberdeen, Foresterhill Health Centre, Westburn Road, Aberdeen, AB25 2AY, UK; 2 University of Aberdeen Business School, Edward Wright Building, Dunbar Street, Old Aberdeen, AB24 3QY, UK; 3 Wigtown Medical Practice, High Vennel Surgery, Wigtown, DG8 9JQ, UK

## Abstract

**Background:**

Patients vary widely when making decisions to consult primary care. Some present frequently with trivial illness: others delay with serious disease. Differences in health service provision may play a part in this. We aimed to explore whether and how patients' consulting intentions take account of their perceptions of health service provision.

**Methods:**

Four focus groups and 51 semi-structured interviews with 78 participants (45 to 64 years) in eight urban and rural general practices in Northeast and Southwest Scotland. We used vignettes to stimulate discussion about what to do and why. Inductive analysis identified themes and explored the influence of their perceptions of health service provision on decision-making processes.

**Results:**

Anticipated waiting times for appointments affected consulting intentions, especially when the severity of symptoms was uncertain. Strategies were used to deal with this, however: in cities, these included booking early just in case, being assertive, demanding visits, or calling out-of-hours; in rural areas, participants used relationships with primary care staff, and believed that being perceived as undemanding was advantageous. Out-of-hours, decisions to consult were influenced by opinions regarding out-of-hours services. Some preferred to attend nearby emergency departments or call 999. In rural areas, participants tended to delay until their own doctor was available, or might contact them even when not on call.

**Conclusion:**

Perceived barriers to health service access affect decisions to consult, but some patients develop strategies to get round them. Current changes in UK primary care are unlikely to reduce differences in consulting behaviour and may increase delays by some patients, especially in rural areas.

## Background

Patients vary widely in their decisions to consult primary care when they are faced with symptoms [1-3]. This has important consequences. On the one hand, patients who delay with symptoms caused by life threatening conditions (like heart disease and cancer) can miss out on life saving treatments [4,5]. On the other, the high level of demand for trivial and self-limiting conditions can swamp scarce primary care and emergency services and affect access for other patients [6].

In a recent study, we set out to explore consulting behaviour in rural and urban areas of Scotland [7]. We identified four main themes. "Personal factors in consulting" were important and included symptom triggers, attitudes to health service use, previous experience, and sanctioning by others. The most important "socio-cultural factor" that differentiated rural and urban areas was a more intense relationship with general practitioners expressed in rural areas, associated with increased visibility of general practitioners in the local community: in urban areas, participants expressed more "consumerist" attitudes to health care. Differences in "access to services" were noted, with rural participants dependent largely on their general practitioner and urban participants more readily accessing hospitals and ambulances. Finally, and perhaps paradoxically, the lack of choice in rural areas seemed to lead to more "complex decision making" in emergencies compared to urban residents; the latter would more readily call an ambulance or attend Accident and Emergency Departments.

Our findings confirmed that patients' decision making processes are complex and a host of factors affect consulting behaviours [8]. They left, however, the general practitioners in the research team asking whether the way we provide primary care services influenced patients' decisions to consult in a meaningful way. Internationally, access to health is regarded as an important factor in consulting and the United Kingdom prides itself on the accessibility of its primary care. Recently provision has changed, however, with most general practices opting out of 24 hour on-call responsibility and increasing roles for nurse advice, consulting and triaging [9]. During routine hours, general practitioners are having their attention diverted by financial incentives from their traditional role responding to new presentations to increasing roles in chronic disease management [10]. Patients requesting new appointments may encounter a variety of planned or unplanned means to manage their demand, including telephone triage and "advanced access" appointment systems, or long waiting times [11-13]. It is unclear what effect these changes are having on the variations in consulting behaviour associated with both unwarranted demand and harmful patient delay.

In this paper, we report further analysis of our above mentioned study [7]. The analysis was conducted as part of our original aims of exploring consulting behaviour in rural and urban areas of Scotland. In this paper, we concentrate on whether and how patients' consulting intentions were related to their perceptions of health service provision.

## Methods

Our methods are described in detail elsewhere [7]. Briefly, eight general practices were approached to take part in the study. We selected practices within two regions of Scotland (the Northeast and Southwest) and ensured representation of small and large general practices in rural and urban locations, because size and location are associated with differences in 1) the accessibility of primary and secondary care, 2) consultation rates [11] and 3) health outcomes [14]. Rural practices were defined as an hour or more drive time from a district general hospital, and outside of market towns [15]: we used distance from a district general hospital as a proxy for restricted access to a range of health and other services. Large practices had three or more GP principals and small ones had one or two.

There are similarities in service provision across UK general practice. In our study, all practices operated appointment systems during routine hours, with some slots kept available for emergencies, home visits for patients deemed unable to travel, and appointments with practice nurses as well as doctors. There are also differences, with longer travelling times in rural areas as well as more limited access to hospital services, and differences in waiting times for appointments. Out of hours, one rural practice provided its own cover, another was part of a small co-operative making use of a community hospital, and the remaining six were part of large co-operatives, whereby calls were triaged by doctors centrally, and consultations arranged at "local" centres (often community hospitals) or by home visit as necessary. Our study coincided with the start of NHS24, a nurse led out-of-hours telephone advice and triage service. This commenced in Northeast Scotland in May 2002, taking over initial telephone contact from the doctors' co-operative, but not its other functions.

Patients between 45 and 64 years old were eligible for the study: we selected this age range as it is the time of life when cancer and heart disease become more common. Each practice was asked to identify and approach for consent a pool of patients (at least 30), mixed in terms of ages (within the eligible range), sex and socio-economic status. Purposive sampling – again to ensure representation from all age, sex and socio-economic groups – was used to select participants. All participants gave written informed consent.

**Figure 1:**
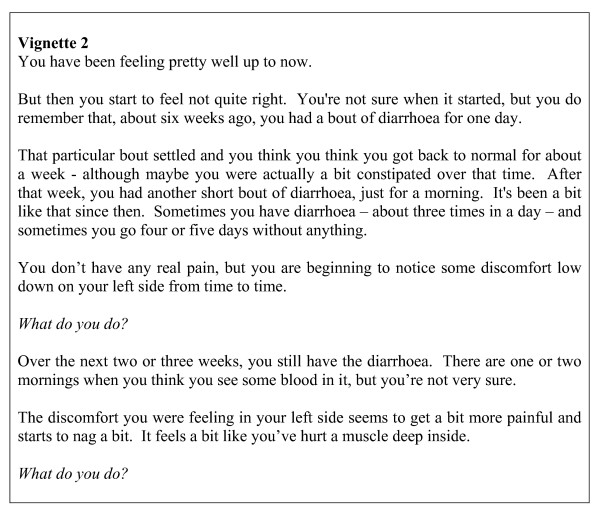
Example of vignette.

We collected data using focus groups and in-depth interviews. Data were collected in 2002. First, we conducted four focus groups facilitated by JF and CG in non-health service locations. We encouraged interaction between urban and rural participants, which we have found a useful way to highlight differences in previous research [15]. We used six audio-taped vignettes, which were designed to stimulate discussion about what participants would do for a range of scenarios. In all of these, the main potential for serious disease was heart disease or cancer, but the likelihood of this varied (from a medical perspective) from low to high (see Figure 1). The vignettes were initially designed by NC, reworded by non-clinical members of the research team, graded in terms of likely severity by six general practitioners from urban and rural locations across Scotland, and piloted with the assistance of a patient participation group (JM). Following preliminary analysis, semi-structured interviews (by CG and two contract interviewers) were held, mainly in participants' homes, to allow us to explore more personal and private responses to vignettes in depth – again methods we have found helpful previously [15]. They were based on the four vignettes that had stimulated most discussion during the focus groups, supplemented by a topic schedule that sought information on health, social, and geographical factors and explored emergent themes.

Focus group dialogue and interviews were audio-taped with participants' permission and transcribed verbatim. The analysis for this paper was undertaken as part of the main analysis for the study. Initial analysis of transcripts was manual [16]. Two researchers read all transcripts independently and other members of the research team read samples. Emerging themes were discussed and consensus reached on an initial coding schedule. On the basis of this schedule, systematic textual analysis was conducted using NVivo software to identify, confirm and develop hypotheses, and note variations and deviant cases. Emergent themes were verified by a group of patients from the Wigtown general practice. In order to explore decision-making processes, individuals' complete texts were re-analysed, using methods similar to conversation analysis, to explore sequences of related dialogue and track recurring and linked attitudes [17]. Ethical approval was obtained from Local Research Ethics Committees in Grampian and Dumfries and Galloway

## Results

### Response rates

From invitations to 330 people, a pool of 117 willing to take part was generated. Twenty-seven participated in four focus groups (14 men and 13 women; 13 registered with rural practices, 14 with urban), which averaged 90 minutes. A further 51 participated in interviews (27 men and 24 women; 26 registered with rural practices and 25 with urban), which lasted between 30 and 90 minutes. Initial analysis of transcripts confirmed that participants had varied access to primary care and other health services. The vignettes had been perceived by the participants to encompass a broad spectrum of illnesses ranging from self-limiting to serious and requiring immediate medical attention. As expected, participants had widely differing consulting intentions, some seeking medical help much earlier than others.

As discussed above, we identified four main themes – we then mapped their relationships with their perceptions of health service provision (Table 1). It was apparent that there were barriers to consulting within all four broad themes. These barriers were complex, both in terms of their effects on decisions to consult and the strategies patients used to obtain appointments. The remainder of this paper is presented, not by "theme", but by considering the impact of patients' perceptions of health services on their general practice consulting intentions. There were clear differences during and out of routine hours.

### How to get an appointment in routine hours

During routine hours, the main "health service" barrier for patients seeking or obtaining an appointment was the perceived waiting time. This was despite all patients believing they would be seen the same day in an emergency. Often, however, participants were unclear whether symptoms warranted an emergency appointment.

**E7 (female; small rural practice) ***"Well for example *[my husband]*had a rash on his nose, and one Thursday morning I phoned up and I says to the receptionist, 'I was wanting to make an appointment for *[my husband].*' 'Is it urgent Madam?' And I says, 'I don't really know – he's got a rash on his nose.' 'Oh,' she says 'If I had a rash on my nose it would be an emergency. Tell him just to come up at 10 o'clock.'"*

If an appointment was sought with a preferred doctor, participants who were registered at small, rural practices reported the shortest waiting times for appointments: those at large, urban practices, the longest ones. The waiting time varied from same day to almost two weeks, depending on the practice at which participants were registered. Knowledge of long waiting times for appointments could generate uncertainty about whether to consult, as illustrated in this focus group discussion.

**C8 (male; small urban practice) ***"There is another aspect here I would think about, is sometimes, well when you phone the doctor, you invariably have to wait a few days and there is this feeling that it is going to go away."*

G1 (male at small rural practice) indicates disagreement.

**C8 ***"Not with yours...? Well, sometimes with mine then it is a few days and there is this feeling that by the time you have your appointment..."*

**D7 (male; large rural practice) ***"..Aye – it is okay*."

**Moderator ***"But if you book this appointment and then you, then it got better, you perceived that it had gone?*"

**C8 ***"I just phone and cancel it, that would be OK, yes."*

And later:

**C8 ***"I think it depends what it is though. If it was something that you thought was like a ... lump, or a tumour or something like that I wouldn't mind how long it took to go and I would definitely want to go and not have that feeling about that I mentioned earlier. It is only if it was something, like, that you might feel was slightly less – more trivial than – a sore throat or something."*

**D7 ***"Just say, this niggly little thing, because it is going to take a few days for you to see a doctor, you put it off?"*

**C8 ***"Yes, you could do, yeah"*

This extract illustrates two further points. First, indecision about whether to consult was only apparent when symptoms were perceived to be mild (in this instance, the vignette described a dry, irritating cough for three weeks and then some pleuritic chest pain). Second, the urban resident's options included either delaying consulting or booking an appointment, despite believing symptoms to be self-limiting, with a view to cancelling later if symptoms improved. The latter was one of several strategies used by urban participants for obtaining appointments. In this situation the patient thought he had time, and was operating a wait and see approach. In others, when patients wanted a quick appointment, their approach depended on assertiveness.

**H30 (male; large urban practice) **responding to vignette describing brief intermittent palpitations: *"I would be insisting that I get an appointment or somebody come out, I would. I would tell them what it was and insist I get somebody."*

One participant had used out-of-hours for a second opinion when not content with his own doctor's treatment.

**B22 ***"I have called out of hours for that, aha, because I have thought 'well, I am no' getting any satisfaction from my own doctor in four years,' I thought, 'I wonder if another doctor...'. And he said 'Go to your own GP. I think you should be referred back'."*

In rural areas, participants also used strategies to obtain appointments, but sought to use influence rather than assertiveness. Only one rural patient talked of insisting on an appointment – a previous city dweller – and, even here, her language was less confrontational.

**F13 (female; large rural practice) **Vignette described possible myocardial infarction at night. She waited to call her doctor the following morning.*"I'd say 'Can I see you today'. And if they said no, I would explain the situation and say 'Look, I've got to see a doctor today because I'm so concerned about this..."'*

More typically and subtly, rural participants believed that their past record of consulting behaviour would be taken into account by their general practice when they asked for an appointment:

**G6 (male; small rural practice) ***"I'm just judging this personally, but I think they probably grade their patients into those that only ring when there's something wrong – like myself – and those that go a little more often."*

**D40 (male; large rural practice) ***".. I think the doctor must suss them oot o'er a period o' time. Say 'Oh to hell, I have had enough o' you, you bugger!' So that is why she *[receptionist]*just takes me right away, 'cause I dinnae *[don't] *pester, ken *[you know]*?"*

Beliefs that patients should use health services responsibly were not restricted to participants from rural areas – statements about this were frequently made in both urban and rural locations – but only rural participants expressed the belief that their general practice would reward this responsible behaviour by seeing them quickly when they needed it. This belief was embedded in the broader theme we mentioned in the introduction – a tendency towards stronger relationships between doctors and patients in rural areas. We have expanded on this previously [7].

**F22 (male; large rural practice) ***"And it's one of those things – it's a bit like the old boy network – if you know someone, they know you, they know that you're not playing around and they will take you seriously. Whereas other people – difficult."*

Although access to appointments in routine hours was considered good by participants from small rural practices, there were instances where this was countered by a lack of choice, for example when patients did not like their general practitioner's attitude, or were embarrassed:

**G21 (female; small rural practice) ***"I don't know... I wouldn't want to compromise the position between the two of us by asking him to give me examinations in relation to female problems. Although, when we had our breasts checked, he did that, but his practice nurse was there and I thought, okay, and I'm sure that his practice nurse would always be there anyway, but I wouldn't want to put him in an awkward position with us knowing each other so well."*

In these situations, options were limited. They may attempt to see the practice nurse or another general practitioner if there was one, but this could involve waiting or travelling.

### How to get seen out-of-hours

The situation out of hours was different. For most participants in the study, out-of-hours care was provided by a centralised service. Many were uncertain about what the formal arrangements were, unless they had experienced them. For most patients, decisions to consult out of hours depended on their assessment of whether or not their symptoms warranted it, but this assessment could depend on their opinion of the service(s) available. Opinions on out of hours services varied. Some were content:

**H16 (male; large urban practice) in focus group -**Vignette described possible myocardial infarction at night *"Yes, well it is the *[out of hours service]*ain't it at that time – you go straight through to them. I would ask for advice, because you know how busy they are and then, well, whether they would ask me to go up and see them, because they usually do."*

But there were also instances, in both rural and urban areas, where participants expressed concern about their local out of hours service and reluctance to use it. A number of reasons were given for this, including a belief they would not be taken seriously, and lack of faith in the person they would speak to.

**F22 (male; large rural practice) **Vignette described brief intermittent palpitations.*"..which brings me back to the point of the NHS phone line business. Well I think it's difficult because unless you are medical you could put the emphasis on the wrong thing. Unless the person is going to click in – and are they going to do it every time? How many calls are they going to get for a start? I mean, it doesn't matter, you can keep on top line for a wee while, but you are going to get tired even if you are the super-matron or something on the phone and the rest of it. No it's, ehm, I'm a little dubious on that ringing up the phone. I think they pump things up on the computer screen – don't they? – and I think it works it out for them. But if the person describes the wrong thing, you could be going the wrong way. It's a bit worrying. No, it would depend. If it was someone close to you, that, you would obviously worry more and maybe you may. But I think if that was me, there, I think I would wait till the Monday."*

**H43 (female; large urban practice) **Vignette described possible myocardial infarction at night *"...I haven't much faith in *[out-of-hours]*I must admit. I've heard too many stories about them. So, if it come to the push I probably would just phone an ambulance. I've heard a few stories that weren't very nice...It probably just depends who's on call...No doubt some of them are really good*..."

The latter extracts show that patients develop strategies to get around the out of hours service. In some areas, often cited alternatives were to phone 999 for an ambulance, or to go straight to a hospital. The following extracts were responses to a vignette describing symptoms that could have been caused by a myocardial infarction at night. Going straight to hospital was attractive to those who lived near them (including community hospitals in rural areas), but not otherwise, as illustrated in this focus group discussion:

**C20 (female; small urban practice) ***"If it was very severe I would get my husband, if he was there, to drive me to the hospital. To go that one step further, I feel that I would make the judgement that it was flu or something or it was something you know like that. But if I thought it was a heart attack coming on – if I suspected it was something that was actually – I wouldn't phone the *[the out-of-hours service]*because I would feel to be honest that I would be told 'Oh, it is flu' or 'it is indigestion'. If I felt in myself it was something like that, I would get myself somewhere quickly."*

**G18 (female; small rural practice) ***"That's not a bad idea but then again it would depend on how far you have got to travel."*

**C20 ***"I am in *[the city]*so."*

**D23 (female, large rural practice) ***"Well I am 34 miles."*

Calling an ambulance was also less common for patients in rural areas. For many participants, this was because of uncertainty about whether they were allowed to do it – *"I didn't realise you could do that!"*. There was also concern about distances and the time it would take for an ambulance to arrive.

**D2 (male; large rural practice) ***"...Let's see noo...I am sure they have come fae *[from] [the town]*doon to here. My dad he got his hip joints and he gid awa' *[went] *to a roup, a farm sale, this afternoon and his hip joint slipped out and he was just in agony. I thought it was *[the town]*the ambulance come fae. It's ridiculous like, but however...that's the worst of staying out in the wilds out here because they speak abut this nine minutes and stuff o' this kind, but that is impossible staying oot here, like*."

For rural participants without a nearby hospital, the only remaining way to avoid calling the out-of-hours service was to wait and call their own doctor during routine hours. This course of action was recounted several times, even when symptoms were perceived to be severe (as in the possible myocardial infarction vignette):

**F11(female; large rural practice**) in focus group *"Eh, no, I would probably have waited until eight o'clock."*

**Moderator ***"Right, okay, why?"*

**F11 ***"Because if it had been as I am led to believe for that length of time, I think I would be very, very frightened. Because that's, you know, the first half of that was everything – hanging out of the window to try and get the air and breathe and it is so frightening."*

**Moderator ***"But you are leaving it till eight?"*

**F11 ***"Yes because I wouldn't want to disturb the doctor. The doctor I want. Not somebody who is coming from *[the town]*which is three quarters of an hour away. Well nearly three quarters of an hour away from me."*

It is unclear how much this tendency to delay was due to their desire to avoid the other options (out of hours service, travelling to hospital, or calling an ambulance) and how much to their desire to see their own general practitioner. The latter had some influence – rural patients preferred to use their own doctor whenever it was perceived that this was "allowed". For example, one rural patient explained why she would have considered calling her general practitioner even when he was not on duty:

**E7 (female; small rural practice)*** "For example, there was one morning no' so awful long ago, there had been nae electricity or anything about here. The lights and everything went out and we noticed a chap from the two bungalows just out of *[village]*on the way in. A guy came through the village on his bike and he went up to the back door of the doctor's. And then we noticed *[GP] *coming out and this was about 7 o'clock in the morning. And the chap next door to him had been having a heart attack. I know that he died anyway, but *[GP]*he still went to it – you ken *[know]*what I mean."*

**Interviewer ***"But people would do that in a situation, would go and knock on his door?"*

**E7 ***"Aye, just went to his back door and he was just out and into his car and away, you ken *[know] *what I mean. It's no' as if - *[GP]*would nae be the kind of folk that would say, 'Oh, we dinnae start 'till..."'*

**Interviewer ***"'I'm not on call just now"'*

**E7 ***"No. I'm no' saying he's never said that, but no' that I know of anyway."*

## Discussion

When faced with unfamiliar symptoms, patients take their perceptions of health service provision into account as they attempt to decide whether, when, and how to consult. We found instances when patients would delay consulting and others when they used various strategies to obtain appointments. These variations in behaviour were present whether the situations encountered were likely (from a medical perspective) to be serious or trivial.

Attempting to study patients' intentions and actions when faced with symptoms is difficult and the approach we used has both strengths and limitations. We relied on patients' accounts of the actions they would take when presented with scenarios as vignettes. Although we cannot be certain that these accounts would translate into the stated actions, previous studies have shown that patients' reactions to vignettes are predictive of their behaviour [18]. The wider relevance of our findings is limited by the range of locations, scenarios, and participants in our study, but our analysis confirmed that our vignettes were perceived as ranging from trivial to serious, our participants differed widely in consulting intentions, and the health services they had access to varied widely, within the range of what is commonly available in the UK. Participants were from rural, urban, affluent and deprived communities, but ethnic minorities were not represented, so need further research. Finally, this paper has concentrated on perceived health service barriers to patients, which was only one factor amongst many that impact on decisions to consult [8]. The lead author is a general practitioner, so our presentation has a general practice perspective, although three of the five authors are non-clinicians and have ensured a balanced approach to interpreting findings.

Some of our findings are in line with previous research, especially those attitudes that were most often expressed by urban residents. In Tyneside, Gallagher et al found that patients used strategies such as being assertive and threatening to call the doctor out in order to obtain quick appointments [19]. In London, Shipman et al found that some patients would elect to attend A&E if they had difficulty making in hours general practice appointments, anticipated delay from out-of-hours services, or wanted a second opinion [20]. Initiatives like "advanced access" are driven by assumptions about this kind of behaviour, and that the strategies patients use to obtain appointments increase demand and inefficiencies, and reduce access for others [12]. Most previous research on patient consulting has, however, been dominated by urban communities. We have found different, almost opposite, strategies predominating in rural areas, where patients believed that, by being undemanding, they would be taken seriously when they did need help. We found this to be driven by the stronger relationship they appeared to have with their general practitioners and other primary care staff. This contrasted with the "consumerist" orientation to health care that prevailed in urban areas. It is possible that these influences on consulting may be limited to the areas we studied, but two points suggest they are more widely transferable. First, our findings were consistent in two populations at opposite ends of Scotland (over 200 miles apart). Secondly, the existence of a different strategic approach to consulting in rural areas is consistent with internationally observed differences in consulting behaviour – patients in rural areas consult primary health care less frequently than urban patients for both trivial and serious conditions and are more likely to call their own general practitioner in emergencies [21-24].

Our findings on the strategies used by rural patients when consulting have not, to our knowledge, been published before. They paint an encouraging picture of strong relationships between patients and general practitioners [7], but also indicate patients' interests in appearing undemanding and preference to wait to see their own general practitioner if symptomatic out of hours. Their beliefs that undemanding behaviour would lead to more expeditious care at times of need have credence when set against those of general practitioners, who believe, with some justification, that they should be alert to serious disease in patients who are infrequent consulters [25]. They are also, however, deterrents against early consultation and may help to explain why patients in rural areas delay longer before calling with acute myocardial infarction and present with more advanced cancer [3,14].

## Conclusion

Our study has shown that patients take their experience of health service provision into account when deciding whether to consult and how to go about it, and this happens in different ways even within a fairly uniform health service (i.e. the NHS). Our finding suggest, for example, that current changes in out-of-hours services (with increasingly centralised services and reliance on nurse triage) may cause more urban patients to divert themselves to emergency departments and more rural patients to delay consulting. Similar variable responses may explain why initiatives like advanced access may appear attractive to some urban practices, but increase workload in others [26]. Our findings suggest that, somewhat paradoxically, patients in rural areas delay longer before presenting with serious disease because of the strong relationships they have with their general practitioner. The challenge remains, however, to find any system of provision that reduces harmful delay in patients who need to be seen, and unwarranted demand from those who do not.

## Competing interests

The author(s) declare that they have no competing interests.

## Authors' contributions

JF, LI, NC and JM were all involved in study design. CG and JF conducted much of the fieldwork. Primary data analysis was conducted by CG and JF, with all authors contributing. NC drafted the paper, which was revised by all co-authors.

**Table 1:** Relationship between participants' perspectives on health service barriers to consulting and the main groups of inductively identified themes.

	**Personal**	**Socio-cultural**	**Access to services**	**Complexity of decision making**
**Routine hours**	Need to take time off work or other activities	Strength of relationship with primary care personnel	Waiting time for appointment	
		Consumerist attitudes	Availability or lack of choice	

**Out of hours**	Attitude to out of hours service	Strength of relationship with primary care personnel	Distance to A&E facility	Knowledge of which service is available
	Knowledge of ambulance availability	Consumerist attitudes		Attitude to which service is appropriate
		Attitude to using ambulances		

## Pre-publication history

The pre-publication history for this paper can be accessed here:


